# Supplementary material for: A probabilistic assessment of the rapidity of PETM onset
Source: Nat Commun. 2017 Aug 25;8:353. doi: 10.1038/s41467-017-00292-2 (PMC5572461; doi:10.1038/s41467-017-00292-2)
Supplement: Supplementary file 1 — Supplementary Information [file 41467_2017_292_MOESM1_ESM.pdf]

File name: Supplementary Information
Description: Supplementary Figures, Supplementary Tables and Supplementary References

File name: Peer Review File
Description:

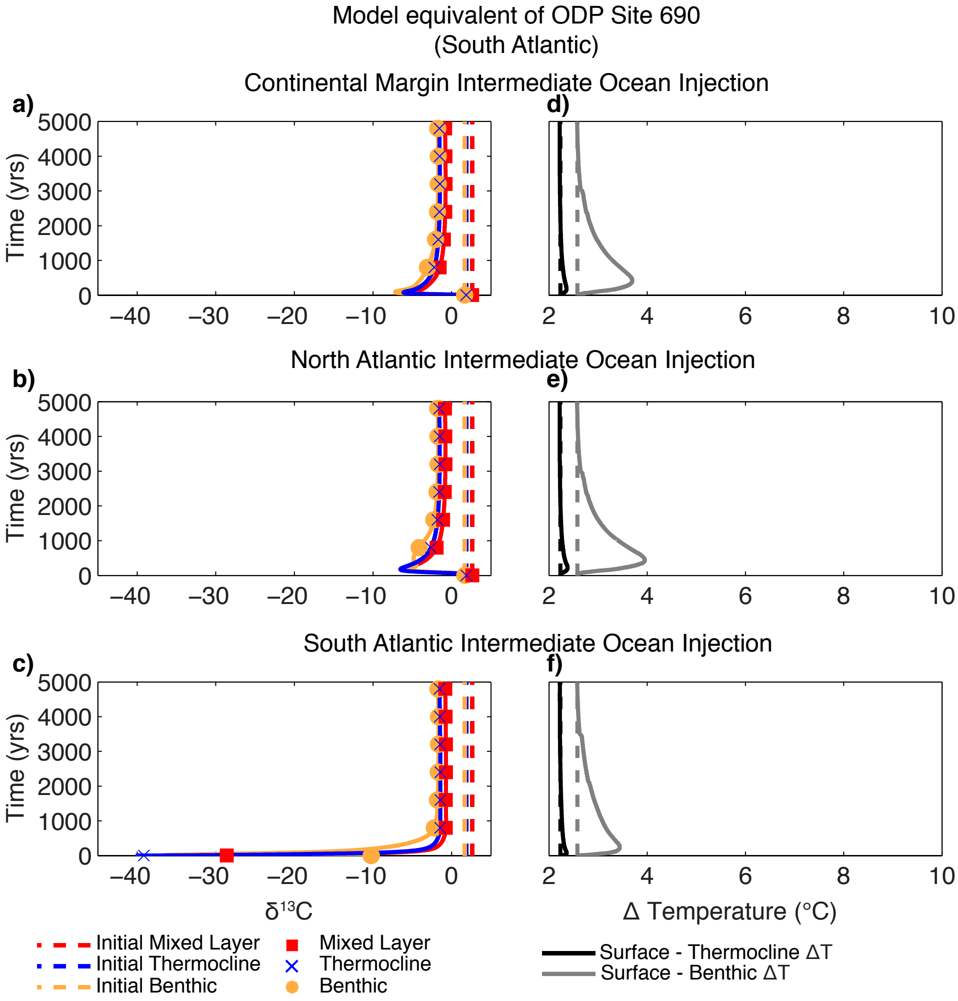

Supplementary Figure 1. Delay timescales at model Site 690 (South Atlantic) for ocean carbon injection experiments. Results shown are for injection of 2275 Pg C with $\delta^{13}C$ of -60‰ to the ocean at intermediate depths (~1000 m) over a single year around all continental margins (a & d), in the North Atlantic only (b & e), and in the South Atlantic only (c & f). (a, b & c) show $\delta^{13}C$ values recorded in the cGENIE mixed layer (red), thermocline (blue), and benthic (orange) ocean layers. Modeled thermocline and benthic depths are 128 m and 3283 m for Site 690. Symbols indicate the record down-sampled at 800-year resolution, consistent with Site 690 data in Ref. 1. (d, e & f) show the change in temperature between the surface and thermocline (black) and surface and benthic (grey). Dashed lines indicate initial temperature differences and solid lines indicate how temperature gradients evolve with time in response to the imposed temperature change.

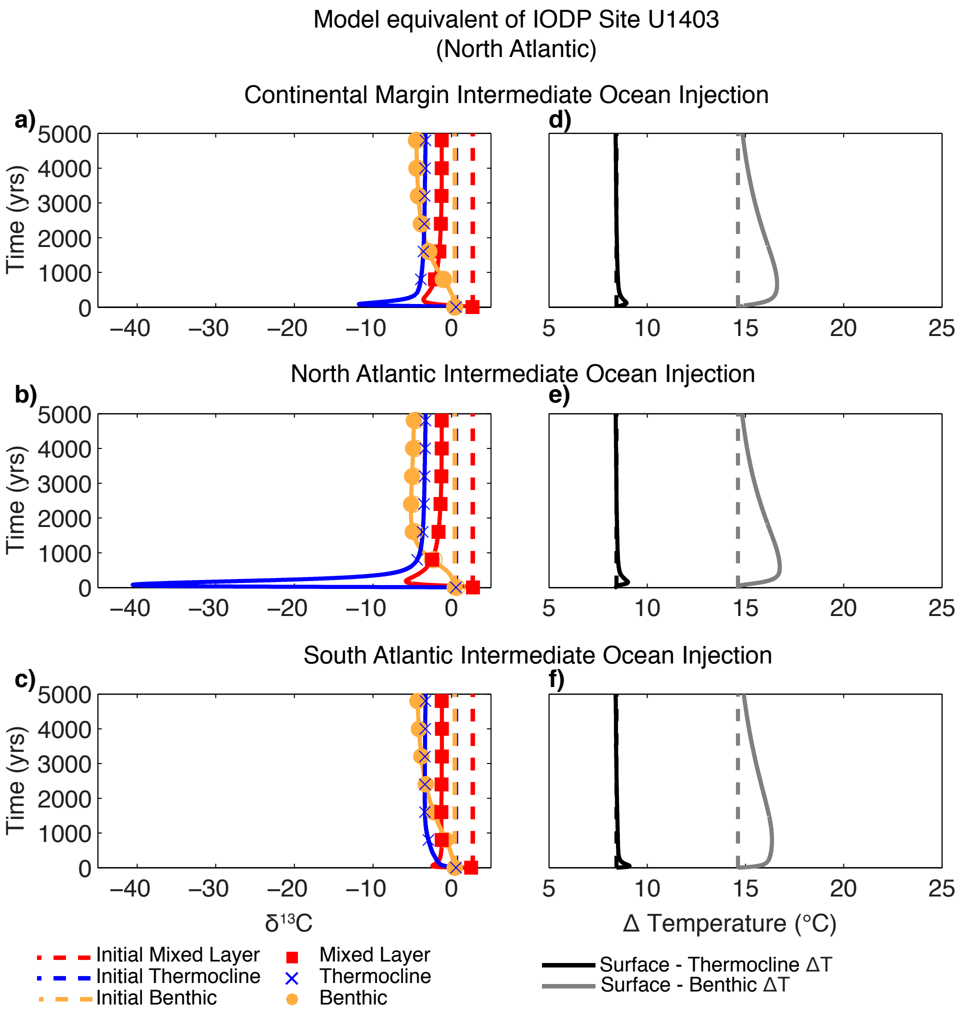

Supplementary Figure 2. Delay timescales at model Site U1403 (North Atlantic) for ocean carbon injection experiments. Results shown are for injection of 2275 Pg C with $\delta^{13}C$ of -60‰ to the ocean at intermediate depths (~1000 m) over a single year around all continental margins (a & d), in the North Atlantic only (b & e), and in the South Atlantic only (c & f). (a, b & c) show $\delta^{13}C$ values recorded in the cGENIE mixed layer (red), thermocline (blue), and benthic (orange) ocean layers. Modeled thermocline and benthic depths are 346 m and 4605 m for Site U1403. Symbols indicate the record down-sampled at 800-year resolution. (d, e & f) show the change in temperature between the surface and thermocline (black) and surface and benthic (grey). Dashed lines indicate initial temperature differences and solid lines indicate how temperature gradients evolve with time in response to the imposed temperature change.

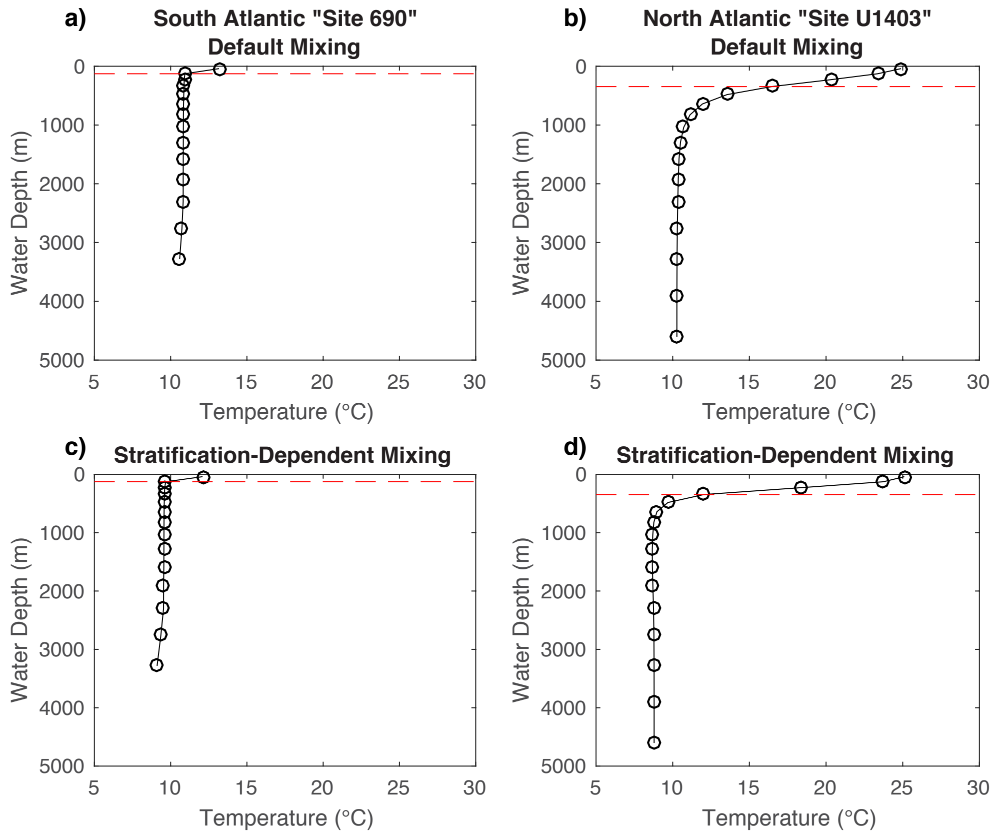

Supplementary Figure 3. Temperature profiles for modeled Site 690 and Site U1403. (a & b) show temperature profiles from model spin-ups with default mixing (i.e. spatially invariant diapycnal diffusivity) and (c & d) show temperature profiles from model spin-ups with stratification-dependent mixing (i.e. spatially variable diapycnal diffusivity as a function of stratification). Dashed red line indicates the thermocline at each site, equal to the ocean depth layer with the maximum temperature gradient.

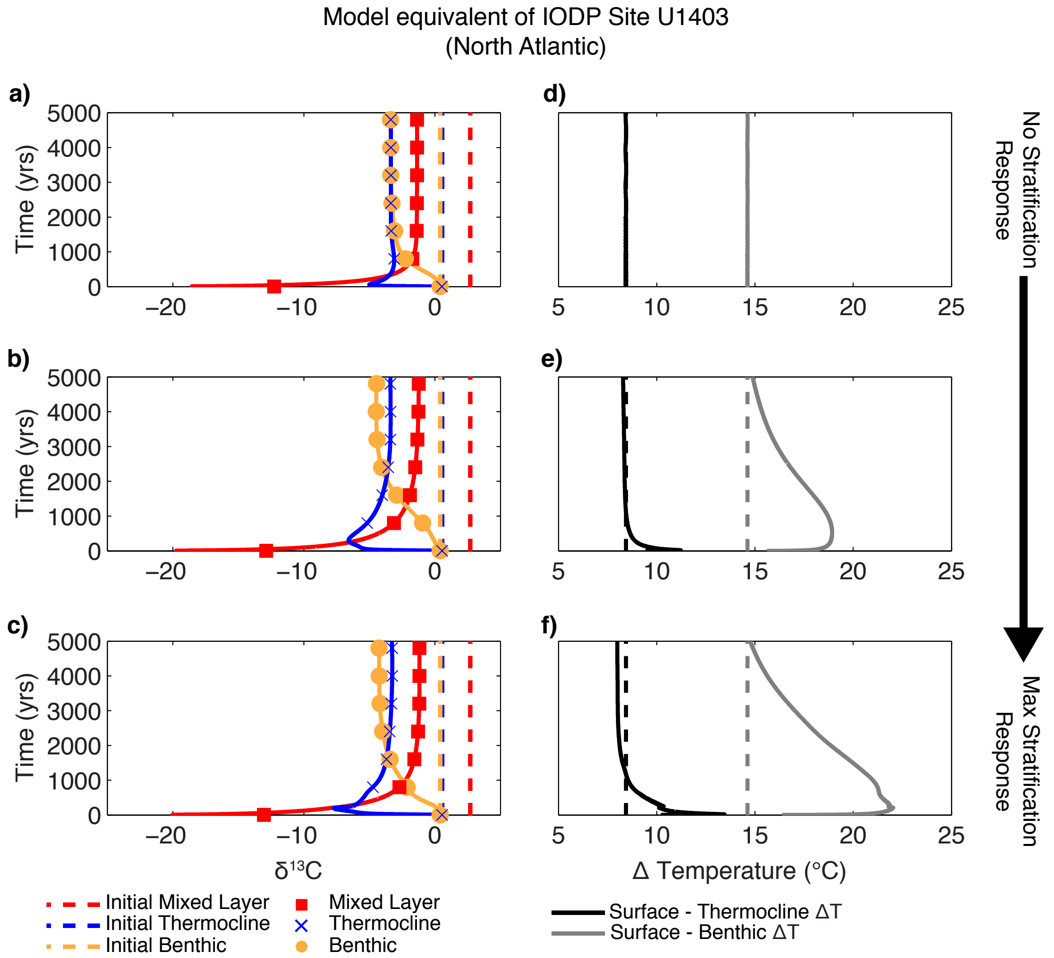

Supplementary Figure 4. Delay timescales at model Site U1403 (North Atlantic) for atmospheric carbon injection experiments. (a, b & c) $\delta^{13}C$ values recorded in the cGENIE mixed layer (red), thermocline (blue), and benthic (orange) ocean layers in response to injection over a single year of 2275 Pg C with $\delta^{13}C$ of -60‰ to the atmosphere. This mass of carbon is sufficient to drive a -4 ‰ global $\delta^{13}C$ excursion based on isotopic mass balance in cGENIE. Modeled thermocline and benthic depths are 346 m and 4605 m for Site U1403. Fixed radiative forcing in each experiment controls the stratification response: at the equivalent of x3 (i.e. no stratification response) (a & d), x10 (b & e) and x25 (c & f) pre-industrial pCO_2. Symbols indicate the record down-sampled at 800-year resolution. (d, e & f) Change in temperature between the surface and thermocline (black) and surface and benthic (grey). Dashed lines indicate initial temperature differences and solid lines indicate how temperature gradients evolve with time in response to the imposed temperature change.

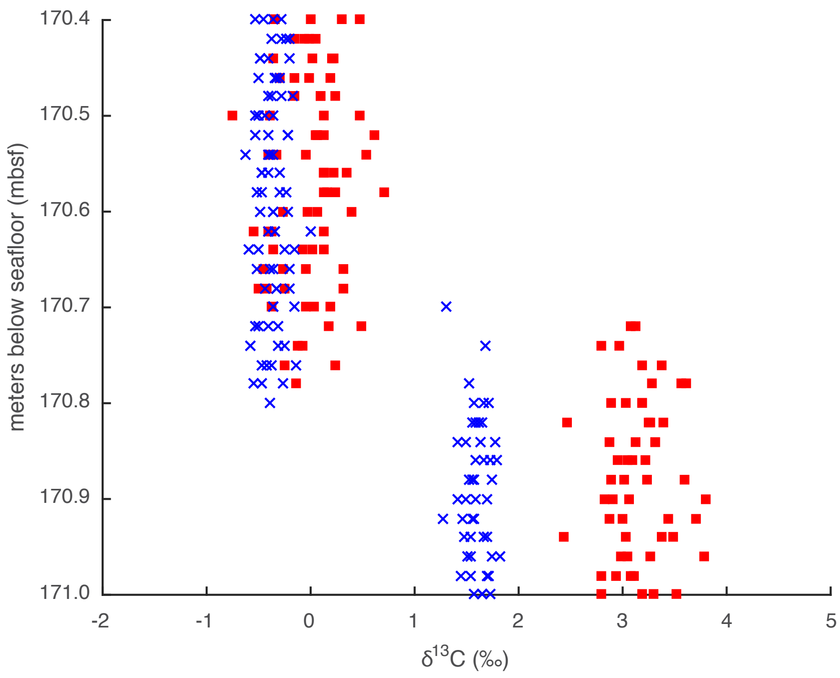

Supplementary Figure 5. With equal abundance changes, surface and thermocline taxa record a synchronous step change in δ^{13}C. Sediment model simulation of δ^{13}C in mixed-layer (red) and thermocline (blue) assuming a 50% decline in the abundance of both species across the CIE onset.

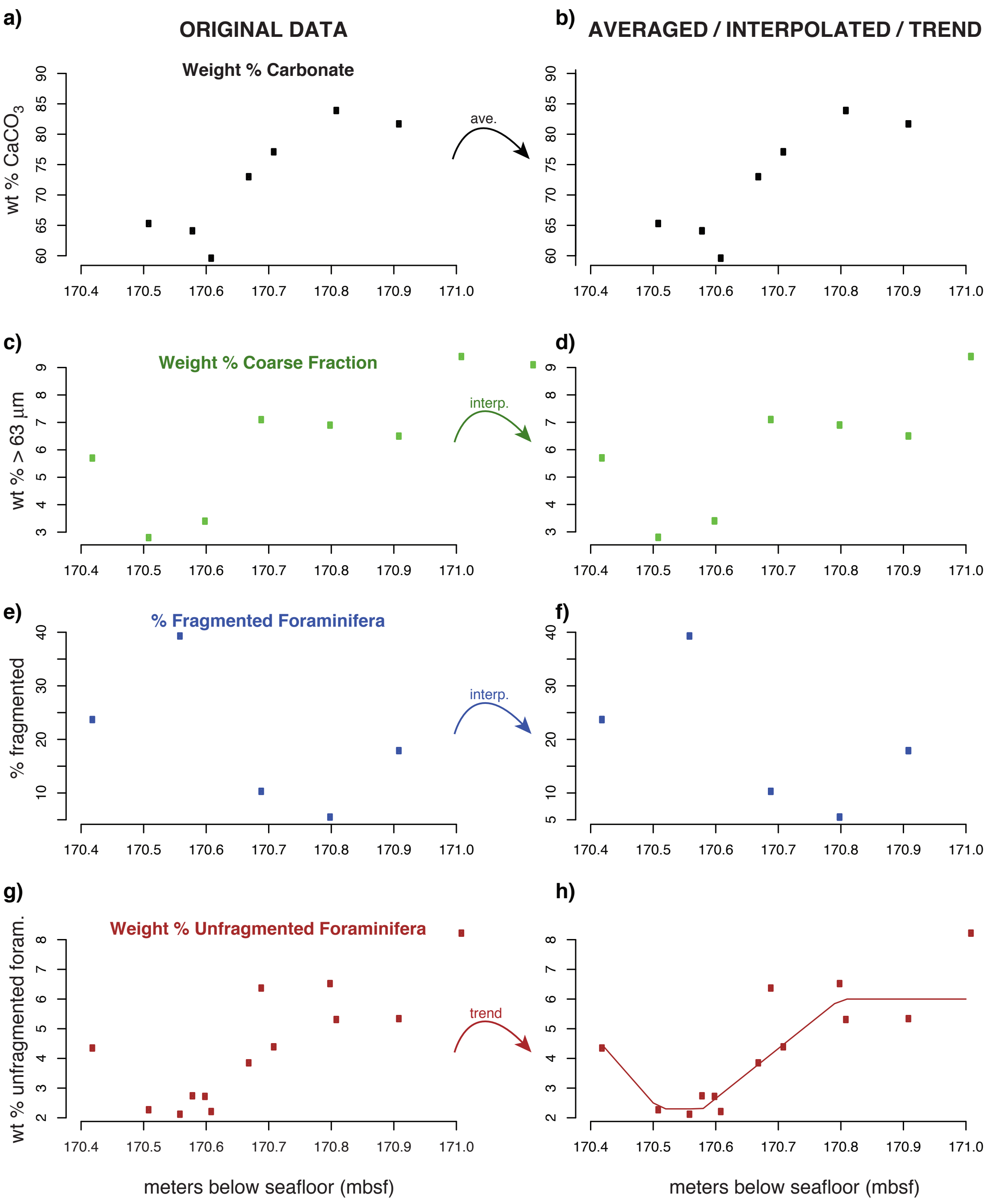

Supplementary Figure 6. Data and calculations to infer the weight % unfragmented foraminifera from the measurements of Ref. 2. (a) Weight % carbonate data did not exist for all sample depths where data was available for weight % coarse fraction (i.e weight % >63 µm) and/or % foraminifera fragmentation. Adjacent weight % carbonate data were averaged to infer the missing values (b; averaged values in open boxes). The linear relationship between weight %

coarse fraction (c) and weight % carbonate was then used to interpolate the weight % coarse fraction data to a similar resolution (d). A similar approach was used to increase the sampling resolution of empirically measured % foraminifera fragmentation (e) to match that of weight % coarse fraction (f). Weight % unfragmented foraminifera (g) was calculated as the product of weight % coarse fraction and 1 - (% fragmented foraminifera). A trend line was fit by eye (h) and used to calculate weight % unfragmented foraminifera matching the generic data (Supplementary Fig. 7).

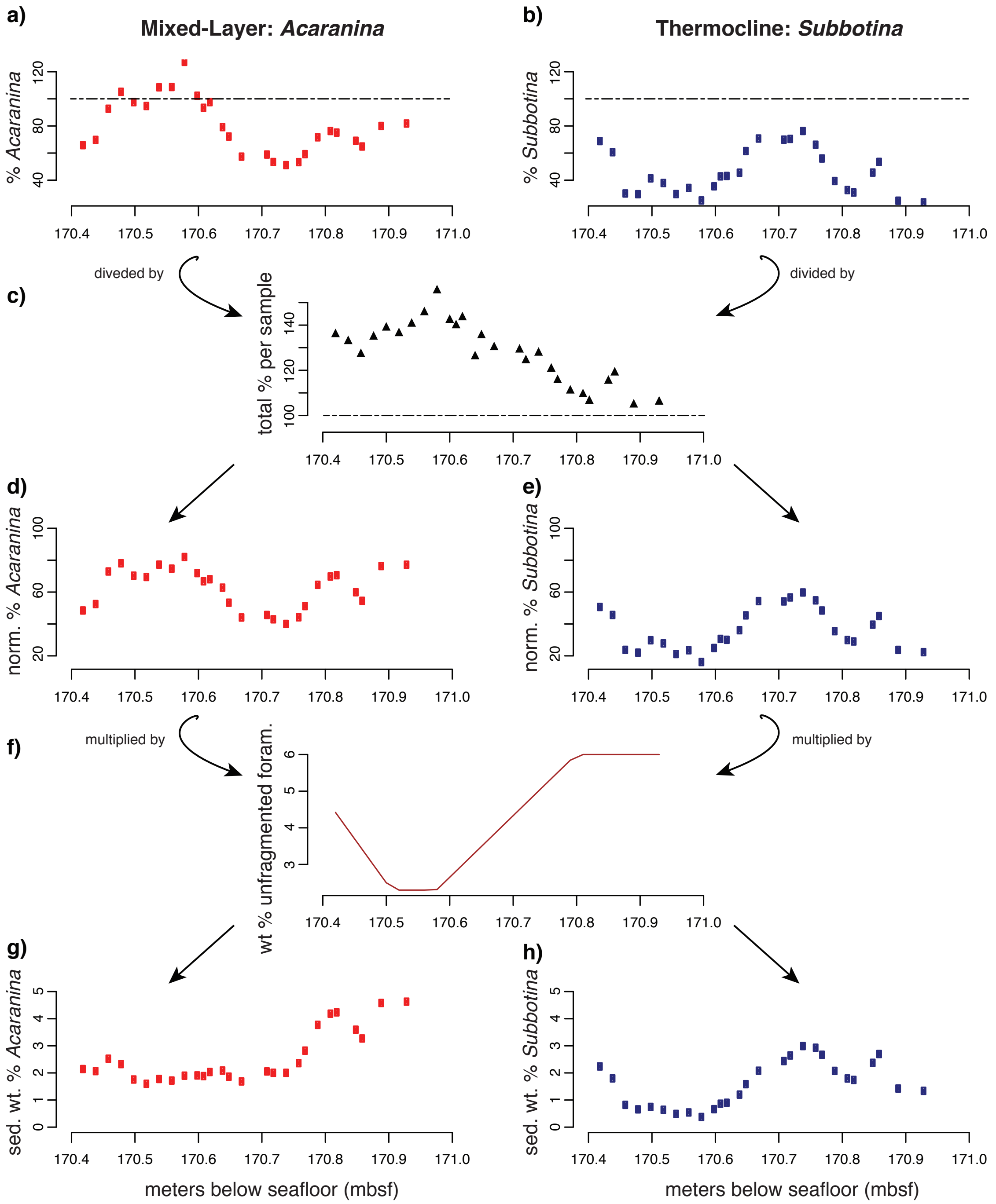

Supplementary Figure 7. Data and calculations to infer the sedimentary % *Acaranina* and sedimentary % *Subbotina*. (a & b) % *Acaranina* and % *Subbotina* were calculated as the sum of the relevant species data from Ref. 3. The sum of all species in each sample (c) always summed to more than 100%, so these sample specific values were used to normalize the initial % *Acaranina* and % *Subbotina* data. Normalized % *Acaranina* and % *Subbotina* (d & e) values were then multiplied by the estimated weight % unfragmented foraminifera (f) to determine the sedimentary weight % *Acaranina* (g) and sedimentary weight % *Subbotina* (h) respectively. The

values (also referred to as sedimentary % *Acaranina* and sedimentary % *Subbotina*) were used as the population targets for the population abundance scenario modeling shown in Fig. 5.

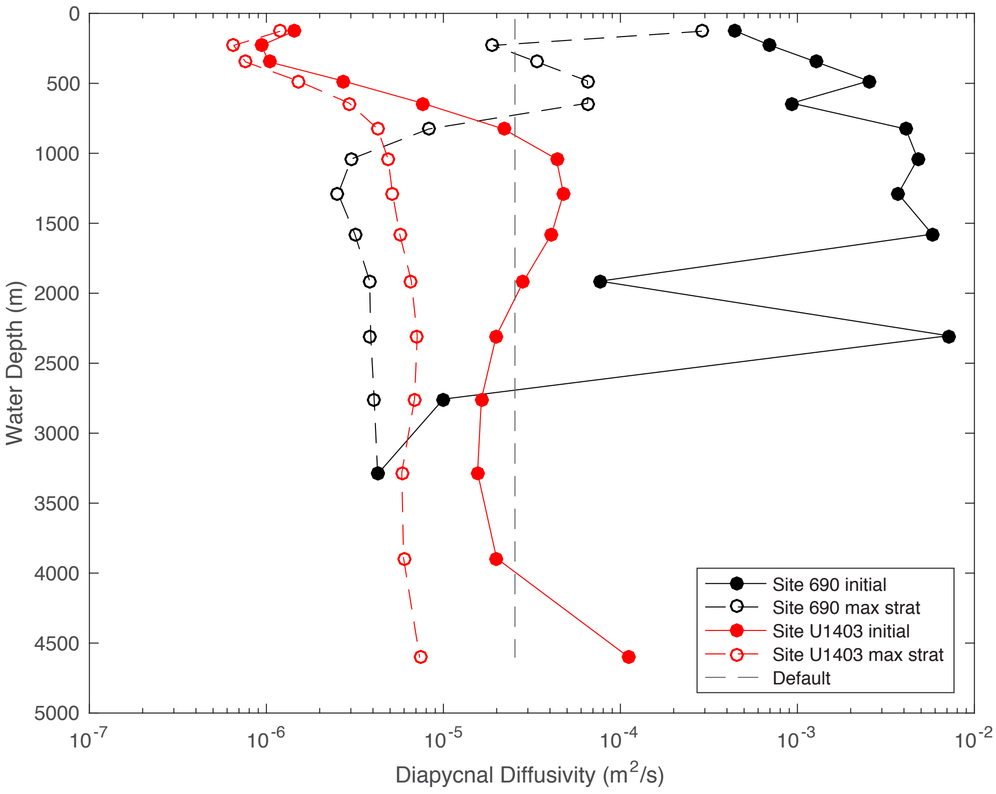

Supplementary Figure 8. Influence of stratification on modeled diapycnal diffusivity at each modeled site using stratification-dependent mixing scheme. Depth profiles of diapycnal diffusivity (m^2 s^{-1}) for model locations corresponding to Site 690 (black) and Site U1403 (red) from the spin-up (solid lines) and in response to maximum stratification (dashed lines). Maximum stratification results are from the x25 pre-industrial CO_2 equivalent radiative forcing experiments at experiment time corresponding to maximum vertical density gradients (year 50).

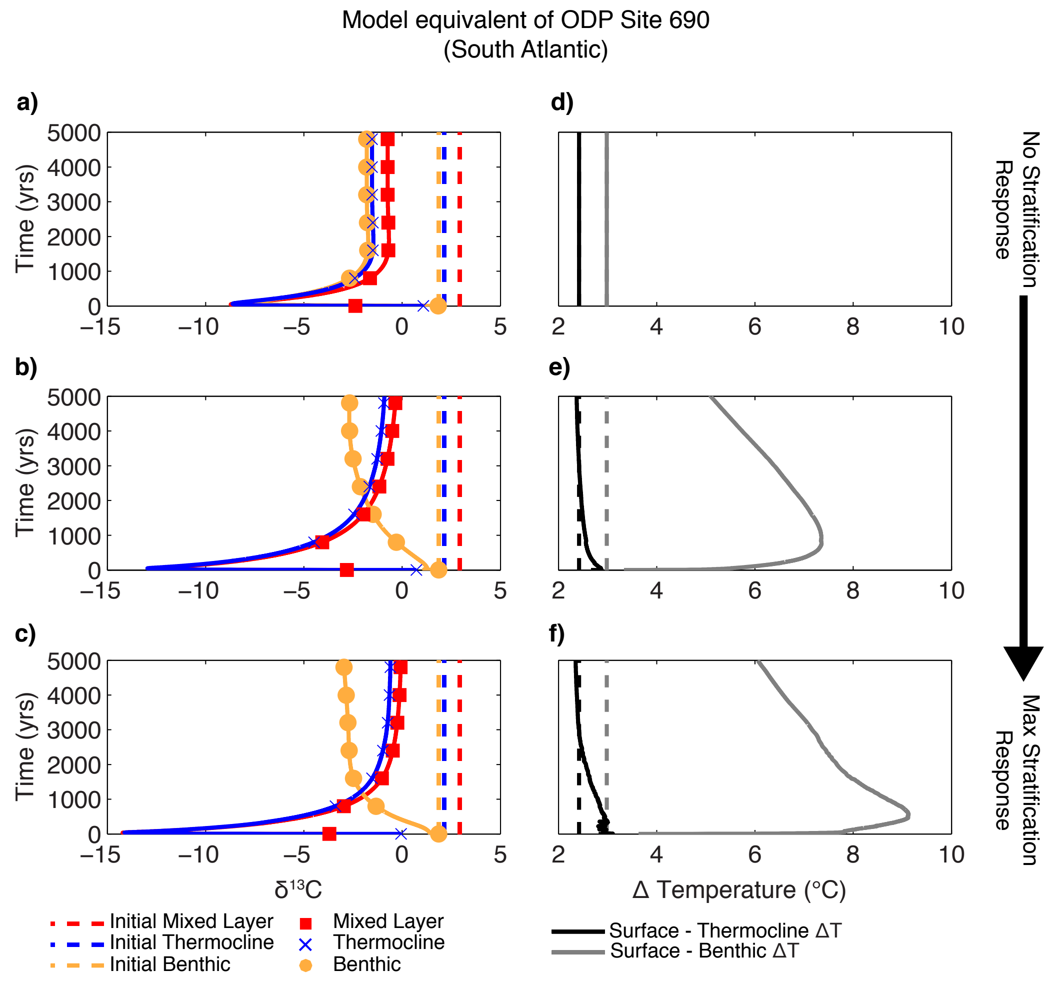

Supplementary Figure 9. Delay timescales at model Site 690 (South Atlantic) for atmospheric carbon injection experiments using stratification-dependent mixing. Experiments shown are identical as those in Fig. 2 except for the incorporation of a stratification-dependent mixing scheme. (a, b & c) $\delta^{13}C$ values recorded in the cGENIE mixed layer (red), thermocline (blue), and benthic (orange) ocean layers in response to injection over a single year of 2275 Pg C with $\delta^{13}C$ of -60‰ to the atmosphere. This mass of carbon is sufficient to drive a -4 ‰ global $\delta^{13}C$ excursion based on isotopic mass balance in cGENIE. Model location is equivalent to ODP Site 690 in the South Atlantic. Modeled thermocline and benthic depths are 128 m and 3283 m for Site 690. Fixed radiative forcing in each experiment controls the stratification response: at the equivalent of x3 (i.e. no stratification response) (a & d), x10 (b & e) and x25 (c & f) pre-industrial pCO_2. Symbols indicate the record down-sampled at 800-year resolution. (d, e & f) Change in temperature between the surface and thermocline (black) and surface and benthic (grey). Dashed lines indicate initial temperature differences and solid lines indicate how temperature gradients evolve with time in response to the imposed temperature change.

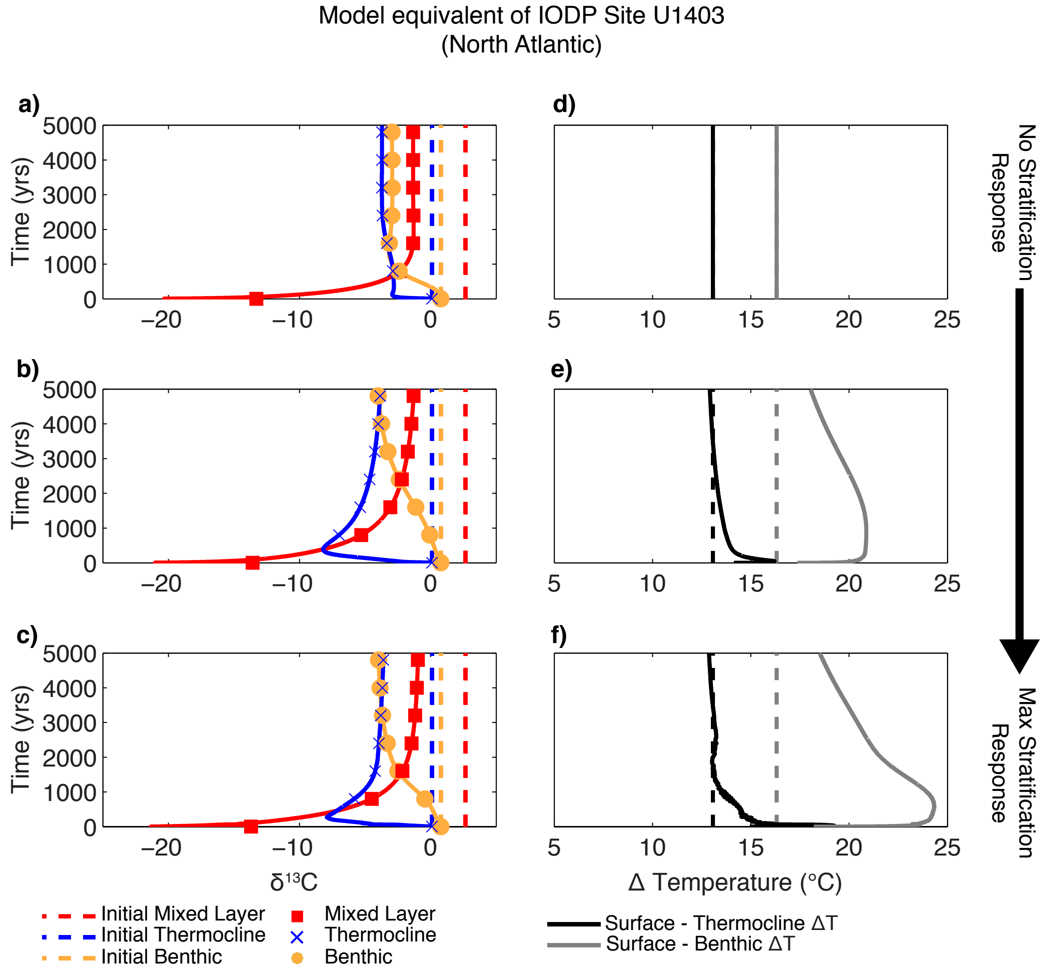

Supplementary Figure 10. Delay timescales at model Site U1403 (North Atlantic) for atmospheric carbon injection experiments using stratification-dependent mixing. Experiments shown are identical as those in Supplementary Fig. S4 except for the incorporation of a stratification-dependent mixing scheme. (a, b & c) $\delta^{13}C$ values recorded in the cGENIE mixed layer (red), thermocline (blue), and benthic (orange) ocean layers in response to injection over a single year of 2275 Pg C with $\delta^{13}C$ of -60‰ to the atmosphere. This mass of carbon is sufficient to drive a -4 ‰ global $\delta^{13}C$ excursion based on isotopic mass balance in cGENIE. Modeled thermocline and benthic depths are 346 m and 4605 m for Site U1403. Fixed radiative forcing in each experiment controls the stratification response: at the equivalent of x3 (i.e. no stratification response) (a & d), x10 (b & e) and x25 (c & f) pre-industrial pCO_2. Symbols indicate the record down-sampled at 800-year resolution. (d, e & f) Change in temperature between the surface and thermocline (black) and surface and benthic (grey). Dashed lines indicate initial temperature differences and solid lines indicate how temperature gradients evolve with time in response to the imposed temperature change.

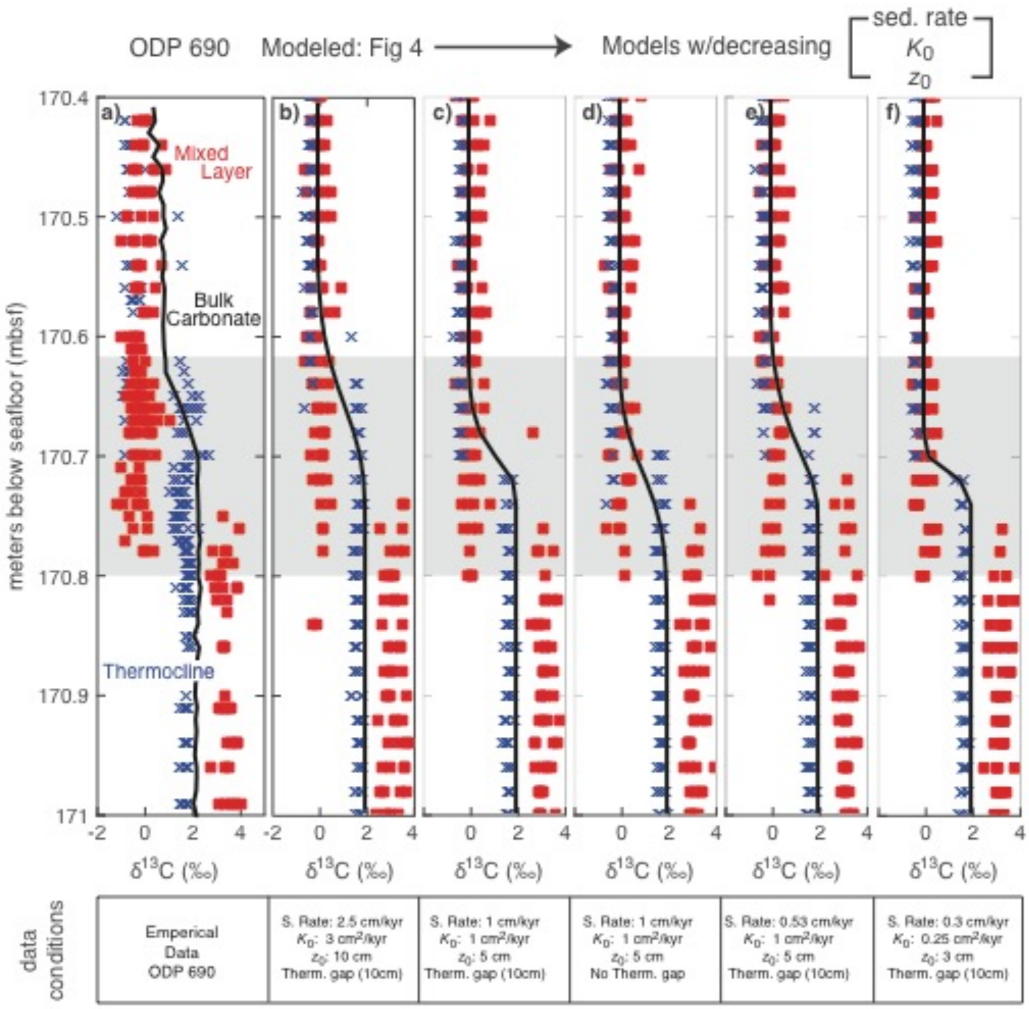

Supplementary Figure 11. The effect of varying sedimentation rate (w_s), maximum diffusivity (K_0), and mixing depth (z_0) on modeled isotopic values. a) Single-foraminifera isotope data from ODP 690 across the PETM exhibit an apparently diachronous step-change in mixed-layer planktonic foraminifera $\delta^{13}C$ (red), thermocline $\delta^{13}C$ (blue), and bulk carbonate $\delta^{13}C$ (black) values from Ref. 1. This diachronous step-change is readily simulated (b; same parameters as in Fig. 4b), assuming a greater change in the abundance of thermocline and bulk carbonate species than mixed-layer species (shown in panel (c), with lines = pre-mixing abundance; symbols= post-mixing abundance). In this scenario there is a synchronous change in stable isotopes ($\delta^{13}C$) in surface and mixed layer depths –it is the combination of bioturbation and unequal abundance change that makes the sedimentary record appear diachronous. Decreasing sedimentation rate (w_s), maximum diffusivity (K_0), and mixing depth (z_0) in step (from c-f, with panel (d) differing from (c) only in the absence of a 10cm gap in thermocline species) generally decreases the apparent diachroneity in the two mixed records. Mixing model parameters all listed at the bottom of the figure.

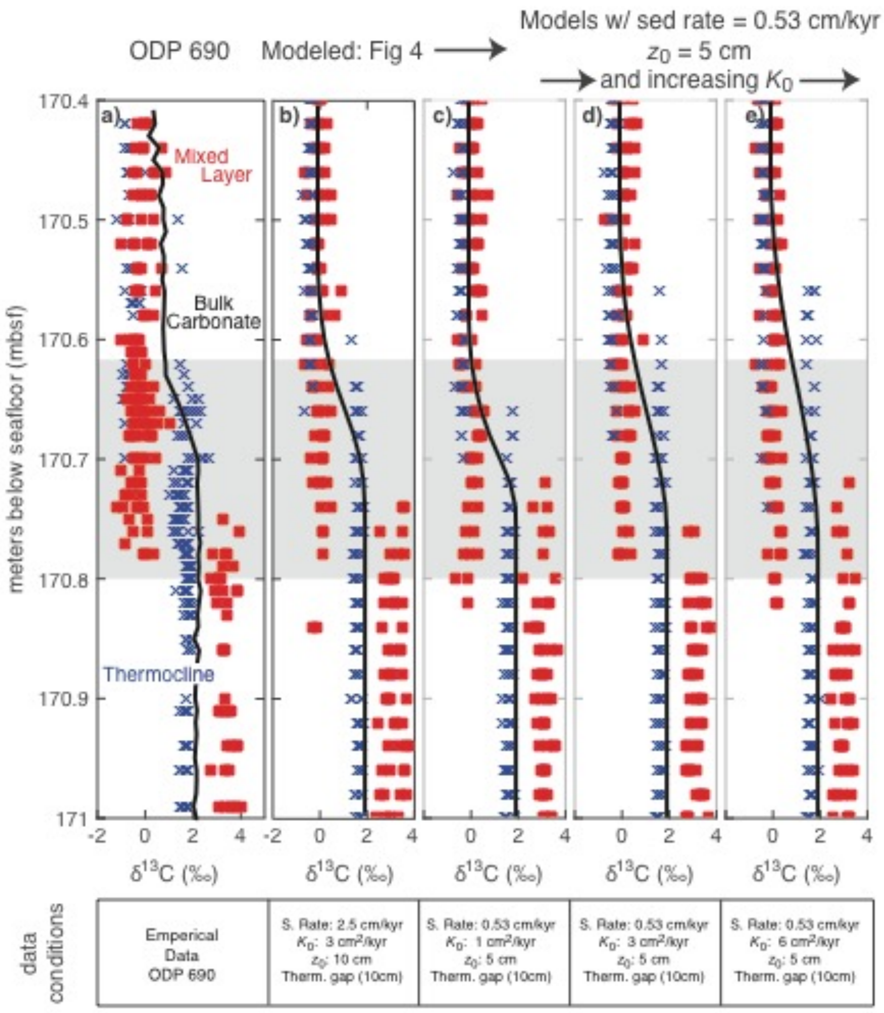

Supplementary Figure 12. The effect of varying maximum diffusivity(K_0) on modeled isotopic values. a) Single-foraminifera isotope data from ODP 690 across the PETM exhibit an apparently diachronous step-change in mixed-layer planktonic foraminifera $\delta^{13}C$ (red), thermocline $\delta^{13}C$ (blue), and bulk carbonate $\delta^{13}C$ (black) values from Ref. 1. This diachronous step-change is readily simulated (b; same parameters as in Fig. 4b), assuming a greater change in the abundance of thermocline and bulk carbonate species than mixed-layer species (shown in panel (c), with lines = pre-mixing abundance; symbols= post-mixing abundance). In this scenario there is a synchronous change in stable isotopes ($\delta^{13}C$) in surface and mixed layer depths –it is the combination of bioturbation and unequal abundance change that makes the sedimentary record appear diachronous. Increasing maximum diffusivity(K_0) from c-e shows that models with low mixing depths (z_0) and sedimentation rates (w_s) like the lowest values estimated for ODP 690, can produce isotope curves like those observed with maximum diffusivities of around 3 cm^2/kyr (d). Mixing model parameters all listed at the bottom of the figure.

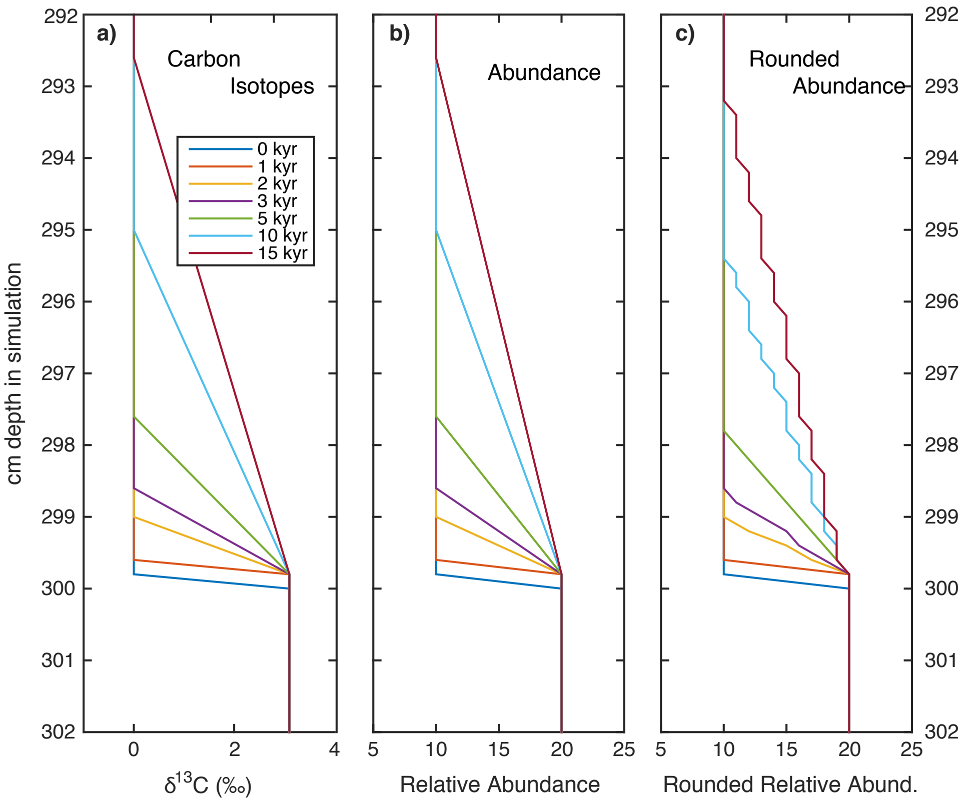

Supplementary Figure 13. CIE onset duration modeling for mixed-layer taxa. a) Isotopic onsets, mean value shown, s.d is 0.3‰ throughout time series, b) and abundance declines, modeled for onset durations of 0,1,2,3,5,10,15 kyr. Sediment mixing model requires integer values for abundance, so abundance was rounded (c) into whole integers before simulated mixing.

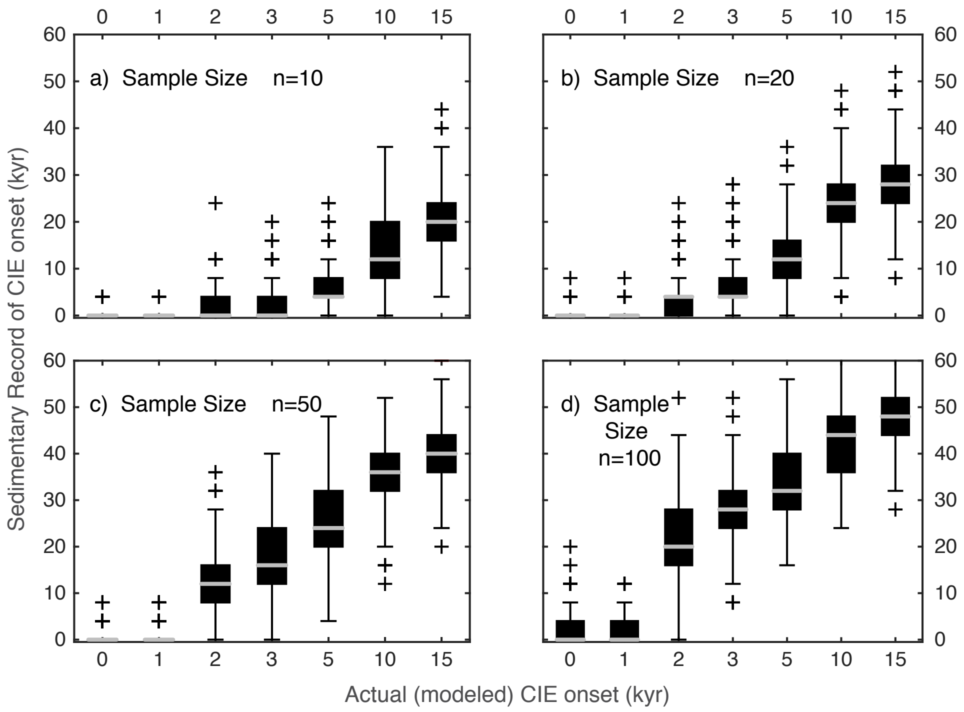

Supplementary Figure 14. Sedimentary records of CIE onset durations at varying sampling intensities. CIE onset durations inferred for sampling intensities of a) n=10, b) n=20, c) n=50 and d) n=100, with all other parameters as in Fig. 6: 2-cm sampling resolution, and sedimentation rates of 0.5 cm/kyr. Detection probabilities optimistically assume that the abundance of the mixed layer species (modeled here) declines from pre- to post-event levels over the CIE onset (see Supplementary Fig. 13). For each box plot, the lower and upper edges correspond to the 25th and 75th percentile, respectively, with the median (50th percentile) indicated as a grey line. Box plot whiskers span ±2.7 s.d, with data lying beyond the whiskers shown as black crosses.

Delay in propagating the $\delta^{13}C$ excursion from the surface to thermocline in cGENIE.

Experiment	Model Site	CIE surf (yr)	CIE thermo (yr)	CIE benthic (yr)	Surf-Thermo Delay Time (yrs)	Surf-Benthic Delay Time (yrs)
**Atm. C input Radforx3 Default mixing**	690	23.5	33.5	53.5	10	30
**Atm. C input Radforx3 Default mixing**	U1403	5.5	57.5	5449.5	52	5444
**Atm. C input Radforx10 Default mixing**	690	41.5	47.5	1739.5	6	1698
**Atm. C input Radforx10 Default mixing**	U1403	4.5	299.5	7699.5	295	7695
**Atm. C input Radforx25 Default mixing**	690	31.5	35.5	2399.5	4	2368
**Atm. C input Radforx25 Default mixing**	U1403	4.5	194.5	9649.5	190	9645
**Ocn C input Default mixing**	690	79.5	73.5	87.5	-6	8
**Ocn C input Default mixing**	U1403	209.5	83.5	6049.5	-126	5840
**N. Atl. C input Default mixing**	690	79.5	73.5	87.5	-6	8
**N. Atl. C input Default mixing**	U1403	179.5	77.5	2249.5	-102	2070
**S. Atl C input Default mixing**	690	1.5	1.5	11.5	0	10/15
**S. Atl C input Default mixing**	U1403	53.5	1979.5	6499.5	1926	6446
**Atm. C input Radforx3 Strat. mixing**	690	41.5	61.5	91.5	20	50
**Atm. C input Radforx3 Strat. mixing**	U1403	4.5	3049.5	1379.5	3045*	1375
**Atm. C input Radforx10 Strat. mixing**	690	43.5	47.5	4299.5	4	4256
**Atm. C input Radforx10 Strat. mixing**	U1403	4.5	384.5	6949.5	380	6945

**Atm. C input Radforx25 Strat. mixing**	690	35.5	39.5	5599.5	4	5564
**Atm. C input Radforx25 Strat. mixing**	U1403	4.5	269.5	5999.5	265	5995
**Ocn. C input Strat. mixing**	690	119.5	104.5	114.5	-15	-5
**Ocn. C input Strat. mixing**	U1403	374.5	99.5	4999.5	-279	4625
**N. Atl. C input Strat. mixing**	690	289.5	279.5	684.5	-10	395
**N. Atl. C input Strat. mixing**	U1403	269.5	81.5	2299.5	-188	2030
**S. Atl. C input Strat. mixing**	690	1.5	1.5	16.5	0	15
**S. Atl. C input Strat. mixing**	U1403	81.5	2149.5	6349.5	2068	6268

Supplementary Table 1. Time of arrival of the minimum $\delta^{13}C$ value in the surface, thermocline, and benthic model ocean layers and difference in arrival time between surface and thermocline and surface and benthic for each cGENIE experiment. Radfor indicates experiments with fixed radiative forcing equivalent to a given level of atmospheric pCO_2 (x3, x10, or x25 pre-industrial). Atm = atmosphere, Ocn = ocean, Atl=Atlantic, Strat=stratification-dependent. See Methods for descriptions of each experiment. *Misleading value because $\delta^{13}C$ declines rapidly but then slowly drifts to an absolute minimum value (see Supplementary Fig. 10).

Generic data from Ref. 3.

Depth (mcd)	% *Acaranina*	% *Subbotina*	total % all species	normal. % *Acaranina*	normal. % *Subbotina*
170.42	65.83	68.84	135.93	48.43	50.64
170.44	69.69	60.62	132.86	52.45	45.63
170.46	92.67	30.17	127.14	72.89	23.73
170.48	105.18	29.63	134.81	78.02	21.98
170.5	97.52	41.32	138.84	70.24	29.76
170.52	94.7	37.88	136.37	69.44	27.78
170.54	108.5	29.71	140.56	77.19	21.14
170.56	108.72	34.23	145.63	74.65	23.5
170.58	127.28	25	155.31	81.95	16.1
170.6	102.27	35.45	142.26	71.89	24.92
170.61	93.37	42.8	139.87	66.75	30.6
170.62	97.54	43.08	143.4	68.02	30.04
170.64	79.14	45.49	126.13	62.74	36.07
170.65	72.19	61.44	135.43	53.3	45.37
170.67	57.33	70.69	130.17	44.04	54.31
170.71	58.9	69.87	129.11	45.62	54.12
170.72	53.36	70.43	124.39	42.9	56.62
170.74	51.08	76.28	127.72	39.99	59.72
170.76	53.3	66.11	120.65	44.18	54.79
170.77	59.17	55.97	115.6	51.19	48.42
170.79	71.62	39.36	110.98	64.53	35.47
170.81	76.27	32.69	109.35	69.75	29.89
170.82	75.1	30.87	106.43	70.56	29
170.85	69.04	45.58	115.3	59.88	39.53
170.86	64.82	53.45	118.96	54.49	44.93
170.89	79.95	24.83	104.78	76.3	23.7
170.93	81.78	23.64	106.06	77.11	22.29

Supplementary Table 2. Species relative abundance data (reported as %) from Ref. 3 and downloaded from Pangaea (https://doi.pangaea.de/10.1594/PANGAEA.842889) were summed to calculate the % *Subbotina*, % *Acaranina* and total % of all species. % *Subbotina* is the summation of data from the columns S. patagonica and Subbotina spp of Ref. 3. % *Acaranina* is the summation of A. praepentacamerata, A. mckannai, A. nitida, A. subsphaerica, A. coalingensis, A. soldadoensis, Acarinina spp. and A. wilcoxensis from Ref. 3. All 14-species listed in the Pangaea data table for Ref. 3 were summed to calculate the total % species for each sample. This total was used to normalize the generic data to a total % for all species of 100%.

Weight % unfragmented foraminifera calculated from data in Ref. 2.

Depth (mcd)	wt % $CaCO_3$	wt % >63 µm	% fragment	int. wt % $CaCO_3$	int. wt % >63 µm	int. % fragment	wt % unfragment. foraminifera
170.31	66.5	9.1	22.3	66.5	9.1	22.3	7.07
170.42		5.7	23.7	65.9	5.7	23.7	4.35
170.51	65.3	2.8		65.3	2.8	19.03	2.27
170.56			39.3	64.7	3.49	39.3	2.12
170.58	64.1			64.1	3.41	19.42	2.74
170.6		3.4		61.85	3.4	20.14	2.72
170.61	59.6			59.6	2.79	20.87	2.21
170.67	73			73	4.62	16.55	3.85
170.69		7.1	10.3	75.05	7.1	10.3	6.37
170.71	77.1			77.1	5.18	15.23	4.39
170.8		6.9	5.5	80.5	6.9	5.5	6.52
170.81	83.9			83.9	6.1	13.04	5.31
170.91	81.7	6.5	17.9	81.7	6.5	17.9	5.34
171.01		9.4		85.55	9.4	12.51	8.22
171.11	89.4	6.1	10.7	89.4	6.1	10.7	5.45
171.21		6.4		81.4	6.4	13.85	5.51
171.31	73.4	5.5	9.2	73.4	5.5	9.2	4.99
171.42		6	10	73.4	6	10	5.4

Supplementary Table 3. An estimate of the weight % unfragmented foraminifera was derived from the weight % carbonate data (wt % $CaCO_3$), weight % coarse fraction data (wt % >63µm), and % fragmented (e.g., foraminiferal fragments) in Ref. 2 from Pangaea (https://doi.pangaea.de/10.1594/PANGAEA.787773), as described in the Methods.

Estimates of sedimentary weight % Acaranina and sedimentary weight % Subbotina.

Depth (mcd)	est. wt % unfragment. foraminifera	sed. wt % *Acaranina*	sed. wt % *Subbotina*
170.42	4.42	2.14	2.24
170.44	3.94	2.07	1.80
170.46	3.46	2.52	0.82
170.48	2.98	2.32	0.66
170.50	2.50	1.76	0.74
170.52	2.30	1.60	0.64
170.54	2.30	1.78	0.49
170.56	2.30	1.72	0.54
170.58	2.31	1.90	0.37
170.60	2.65	1.91	0.66
170.61	2.82	1.88	0.86
170.62	2.99	2.03	0.90
170.64	3.32	2.09	1.20
170.65	3.49	1.86	1.58
170.67	3.83	1.69	2.08
170.71	4.50	2.05	2.44
170.72	4.67	2.00	2.64
170.74	5.01	2.00	2.99
170.76	5.34	2.36	2.93
170.77	5.51	2.82	2.67
170.79	5.85	3.77	2.07
170.81	6.00	4.19	1.79
170.82	6.00	4.23	1.74
170.85	6.00	3.59	2.37
170.86	6.00	3.27	2.70
170.89	6.00	4.58	1.42
170.93	6.00	4.63	1.34

Supplementary Table 4. Estimates of weight % unfragmented foraminifera were multiplied by the normalized % *Acaranina* and normalized % *Subbotina* from Supplementary Table 2 to determined the sedimentary weight % *Acaranina* and sedimentary weight % *Subbotina* using the trend line of weight % unfragmented foraminifera shown in Supplementary Fig. 10.

Supplementary References:

1 Thomas, D. J., Zachos, J. C., Bralower, T. J., Thomas, E. & Bohaty, S. Warming the fuel for the fire: Evidence for the thermal dissociation of methane hydrate during the Paleocene-Eocene thermal maximum. *Geology* **30**, 1067-1070, doi:10.1130/0091-7613(2002)030<1067:wtfftf>2.0.co;2 (2002).

2 Kelly, D. C., Nielsen, T. M. & Schellenberg, S. A. Carbonate saturation dynamics during the Paleocene–Eocene thermal maximum: Bathyal constraints from ODP sites 689 and 690 in the Weddell Sea (South Atlantic). *Marine Geology* **303**, 75-86, https://doi.org/10.1016/j.margeo.2012.02.003 (2012).

3 Kelly, D. C. Response of Antarctic (ODP Site 690) planktonic foraminifera to the Paleocene–Eocene thermal maximum: faunal evidence for ocean/climate change. *Paleoceanography* **17**, doi:10.1029/2002PA000761 (2002).
